# Carnosine scavenging of glucolipotoxic free radicals enhances insulin secretion and glucose uptake

**DOI:** 10.1038/s41598-017-13649-w

**Published:** 2017-10-17

**Authors:** Michael J. Cripps, Katie Hanna, Charlie Lavilla, Sophie R. Sayers, Paul W. Caton, Craig Sims, Luigi De Girolamo, Craig Sale, Mark D. Turner

**Affiliations:** 1 Interdisciplinary Biomedical Research Centre, School of Science and Technology, Nottingham Trent University, Clifton, Nottingham, NG11 8NS UK; 2 Diabetes and Nutritional Sciences Division, King’s College London, London, United Kingdom SE1 1UL; 3 Sport, Health and Performance Enhancement (SHAPE) Research Centre, School of Science and Technology, Nottingham Trent University, Clifton, Nottingham, NG11 8NS UK

## Abstract

The worldwide prevalence of diabetes has risen to 8.5% among adults, which represents a staggering rise in prevalence from 4.7% in 1980. Whilst some treatments work by increasing insulin secretion, over time their effectiveness decreases. We aim to increase insulin secretion by developing strategies that work through mechanisms independent of current treatment options. Isolated CD1 mouse islets, INS-1 pancreatic β-cells, or C2C12 mouse myotubes were incubated in standard tissue culture media, or media supplemented with 28 mM glucose, 200 μM palmitic acid, and 200 μM oleic acid as a cellular model of diabetic glucolipotoxicity. Intracellular reactive species content was assayed using 2′,7′-dichlorofluorescein diacetate dye, inducible nitric oxide synthase levels determined by Western blot, 3-nitrotyrosine and 4-hydrpxnonenal both assayed by ELISA, insulin secretion quantified using ELISA or radioimmunoassay, and glucose uptake determined through 2-deoxy glucose 6 phosphate luminescence. Our data indicate that carnosine, a histidine containing dipeptide available through the diet, is an effective scavenger of each of the aforementioned reactive species. This results in doubling of insulin secretion from isolated mouse islets or INS-1 β-cells. Crucially, carnosine also reverses glucolipotoxic inhibition of insulin secretion and enhances glucose uptake into skeletal muscle cells. Thus, carnosine, or non-hydrolysable carnosine analogs, may represent a new class of therapeutic agent to fight type 2 diabetes.

## Introduction

In 2011 it was estimated that there were 347 million people worldwide living with diabetes^1^. However the incidence of diabetes continues to grow at an alarming rate, with the figure in 2030 projected to be more than double that reported in 2000 ^2^. More than 90% of these individuals have type 2 diabetes (T2D), a disease characterized by peripheral insulin resistance and pancreatic β-cell dysfunction. There are a limited number of options to treat T2D, and oral and injectable medications often become less effective over time. Thus, there is an urgent need to better understand the causes of diabetes, and to identify new targets for the development of novel treatment strategies.

T2D is characterized by a failure to control glucose homeostasis, and numerous diabetic complications are attributable to exposure of tissues to high glucose. Although the cause of T2D is multifactorial, nearly 80% of all people with T2D are also obese. This suggests that obesity may play a central role in the progression from normal glucose tolerance to overt T2D. In particular, patients with T2D typically have high circulating levels of palmitic and oleic fatty acids, which can, in turn, mediate the generation of oxidative stress, a mechanism that has been proposed to contribute to diabetes pathophysiology. However, the reality is likely to be more complex than simply scavenging of reactive oxygen species (ROS), as some anti-oxidants potently inhibit glucose-stimulated insulin secretion, not enhance it^3^.

Promising reports have recently emerged on the positive effect of carnosine (β-alanyl-_L_-histidine), a dipeptide synthesised from β-alanine and histidine, in lowering fasting plasma glucose^4,5^. However, despite the known damaging effect of high glucose^6^ and glucolipotoxicity^7^ upon insulin secretion there is a surprising absence of literature investigating carnosine actions on the pancreas. Data presented herein details the protective action of carnosine against glucolipotoxic reactive species generation in both pancreatic β-cells and myotubes. We report, for the first time, direct evidence of the beneficial impact this has upon insulin secretion, and on glucose uptake into skeletal muscle cells.

## Results

### Carnosine is an Effective Scavenger of Reactive Species in Pancreatic β-cells

**Figure 1:**
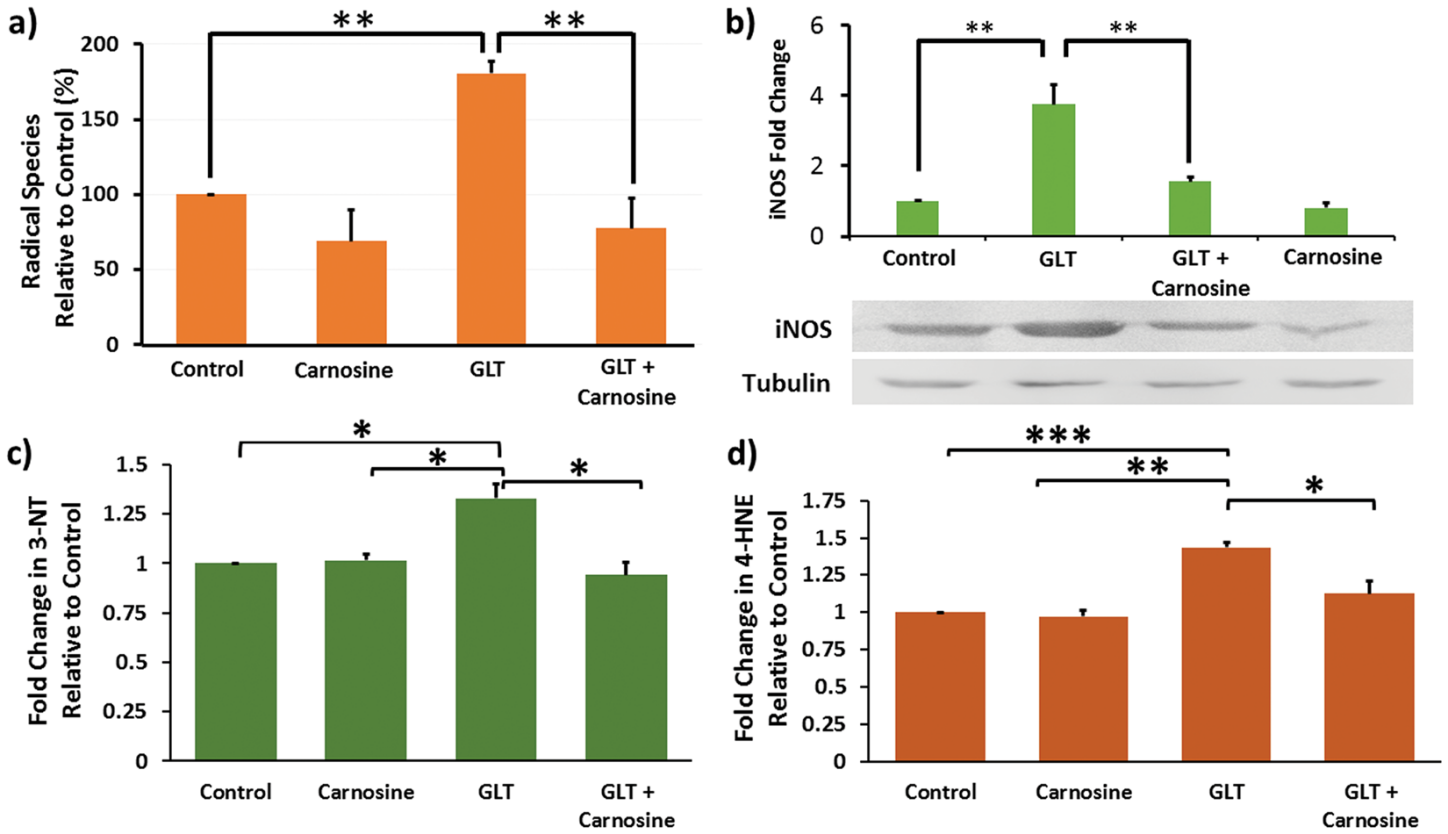
INS -1 cells were cultured in RPMI-1640 media, or RPMI GLT media for 5 days. (**a**) Cells were then incubated for 1 h with media supplemented ± 10 mM carnosine. 20 µM 2′,7-dichlorofluorescin diacetate was then loaded for 1 h and ROS detection measured via fluorescence with excitation and emission of 495 nm and 530 nm. ROS is expressed as percentage change relative to control. N = 4 (**b**) Protein was separated by SDS-PAGE, transferred to nitrocellulose and detected using anti-iNOS primary antibody. Full length blots can be found as Supplementary Figure 1. Protein expression level is expressed as fold change in iNOS relative to control. (**c**) 3-NT formation was determined by ELISA (Abcam), with absorbance measured at 450 nm. (**d**) 4-HNE formation was determined by ELISA (Abexxa) and absorbance measured at 450 nm. In all cases data are expressed as mean ± SEM from 3 or more independent experiments. *p < 0.05 **p < 0.005 ***p < 0.0005, with absolute values as stated in the main text.

Carnosine has recently been shown to be an effective scavenger of reactive carbonyl species (RCS) in a mouse model of diabetic nephropathy^8^. We therefore sought to determine whether carnosine had the ability to scavenge reactive oxygen and nitrogen species (RONS) that are the building blocks for RCS generation in pancreatic β-cells. INS-1 β-cells were cultured for 5 days in Roswell Park Memorial Institute-1640 (RPMI-1640) media, or RPMI supplemented with 28 mM glucose and 200 μM palmitic acid and 200 μM oleic acid (GLT media), including a final 1 h incubation with or without 10 mM carnosine added to the media. 2′,7′-dichlorofluorescein diacetate (DCFDA), a cell permeant fluorogenic dye that measures hydroxyl, peroxyl and other ROS, was then added to cells in order to determine the amount of ROS present in each condition (Fig. 1a). Incubation in GLT media resulted in a significant increase of 80.4 ± 8.2% in ROS (*p* = 6.3 × 10^−5^). However when carnosine was added to GLT media this increase was completely reversed, indicating that carnosine is an effective scavenger of ROS in β-cells. This finding is not the result of altered β-cell viability and glucolipotoxic cell death, as in INS-1 cells treated for 5 days with GLT media we observed 112.23+/−7.03% cell viability.

In order to determine whether carnosine was as effective at scavenging reactive nitrogen species (RNS) we similarly incubated INS-1 cells +/− GLT media+/−carnosine. Cells were lysed, proteins separated by sodium dodecyl sulphate polyacrylamide gel electrophoresis (SDS-PAGE), then transferred to nitrocellulose and immunoblotted with antibody against inducible nitric oxide synthase (iNOS). Band intensity was quantified by densitometry and GLT shown to cause a 3.7 ± 0.6 fold upregulation (*p* = 0.0075) in iNOS expression (Fig. 1b). Addition of carnosine to GLT media showed carnosine is also able to neutralize formation of RNS, inhibiting the GLT upregulation of iNOS by 79.1 ± 4.7% (*p* = 0.017).

As superoxide and NO species were shown to be elevated (Fig. 1a,b) by GLT media, and these species are able to combine to form peroxynitrite, we next sought to determine 3-nitrotyrosine (3-NT) levels in pancreatic β-cells, as this is a marker of cell damage and inflammation that is driven by peroxynitrite. As can be seen (Fig. 1c) GLT results in a 33.0+/−7.4% increase in 3-NT species. Crucially, carnosine is able to completely prevent 3-NT adduct formation (p = 0.0076). Similarly we also sought to determine the effect of GLT on 4-hydroxynonenal, (4-HNE) species generation, an α,β-unsaturated hydroxyalkenal that is produced by lipid peroxidation in β-cells. In this case exposure to GLT media resulted in a 43.46+/−3.43 increase in 4-HNE, with carnosine again able to completely prevent adduct formation (p = 0.026).

### Carnosine Increases Insulin Secretion

**Figure 2:**
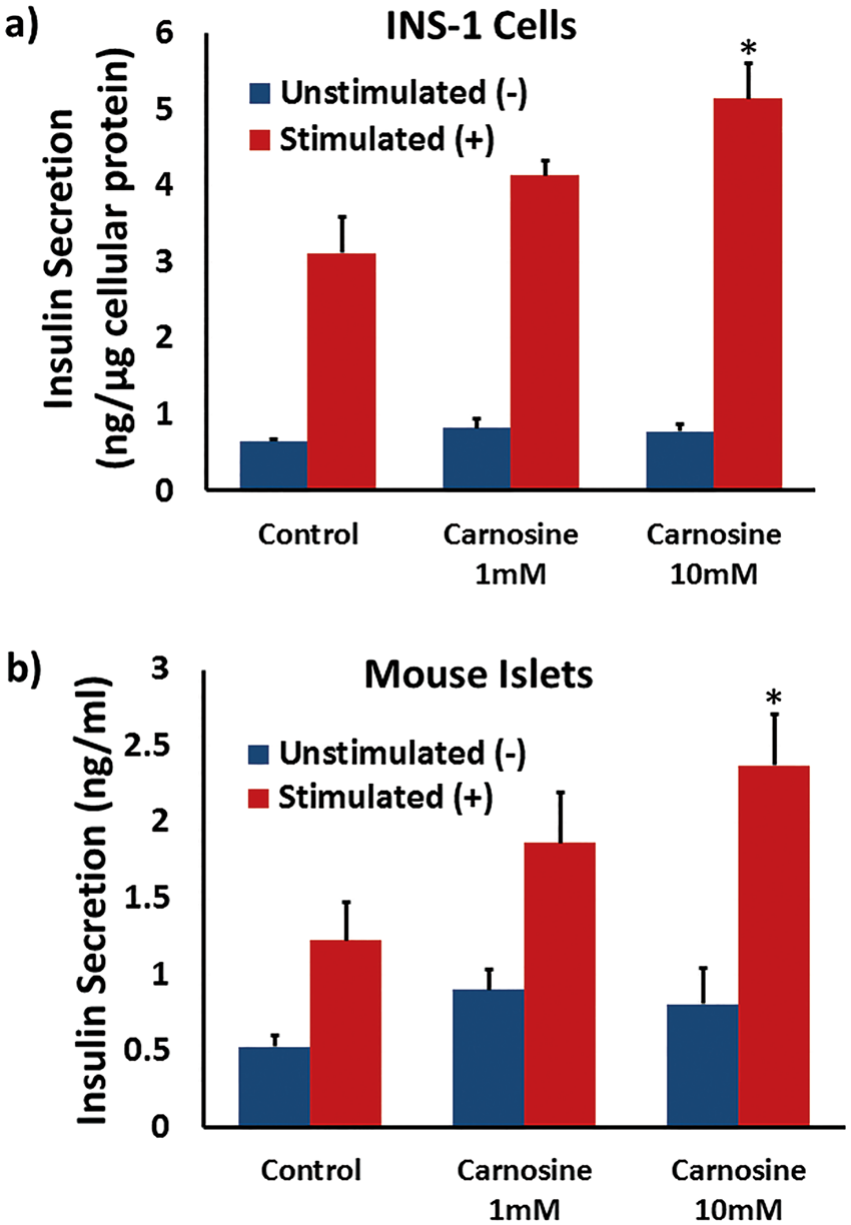
(**a**) INS-1 cells were cultured in RPMI-1640 media, or media supplemented with carnosine, for 5 days. Insulin secretion was determined by ELISA assay following incubation ± secretagogue cocktail for 2 h [(−) blue, (+) red], with data normalized to cellular protein content. Data are expressed as mean ±SEM from 3 independent experiments. (**b**) Islets were isolated from CD1 mice, then cultured in RPMI-1640 media, or media supplemented with carnosine, for 48 h. Islets were incubated in 2 mM [(−) blue] or 20 mM [(+) red] glucose for 1 h as indicated and insulin secretion determined by radioimmunoassay. Data are expressed mean ± SEM from a minimum of 6 independent experiments. *p < 0.05 compared to control stimulated samples, with absolute values as stated in the main text.

Given the potent scavenging effect of carnosine against RONS we sought to determine whether long-term treatment with carnosine might therefore prove beneficial to β-cell function, and insulin secretion in particular. INS-1 cells were incubated for 5 days in RPMI-1640 media, or RPMI supplemented with either 1 mM or 10 mM carnosine, concentrations that are both within the physiological range found in carnosine-sensitive tissues such as skeletal muscle^9,10^. Cells were washed, then incubated in Krebs-Ringer buffer (KRB) supplemented with the indicated concentration of carnosine+/−secretagogue cocktail for 2 h. Insulin secretion was quantified by ELISA (Fig. 2a), and showed a moderate increase in secretagogue-stimulated insulin secretion of 34.6 ± 8.1% at 1 mM carnosine. There was however a significant increase in insulin secretion of 77.2 ± 18.7% at 10 mM carnosine (*p* = 0.018).

INS-1 cells are a well characterised β-cell-line that offer a robust insulin secretory profile. However as transformed β-cell lines are not always fully representative of primary β-cell biology it is important to determine whether these findings are indicative of whole animal physiology. Therefore islets were isolated from CD-1 mice and cultured in RPMI media, or media supplemented with either 1 mM or 10 mM carnosine, for 48 h, then spun down and incubated in KRB supplemented with 2 mM or 20 mM glucose for 1 h. Insulin secretion was determined with radioimmunoassay (Fig. 2b) and showed a moderate increase in glucose-stimulated insulin secretion of 39.1 ± 47.8% at 1 mM carnosine. There was however a statistically significant increase in insulin secretion of 226 ± 49.3% at 10 mM carnosine (*p* = 0.025). Therefore 10 mM carnosine is a concentration that offers a potent enhancement of stimulated insulin secretion, both in tissue culture β-cells and primary islets.

### Carnosine Reverses Damaging Glucolipotoxic Inhibition of Insulin Secretion

**Figure 3:**
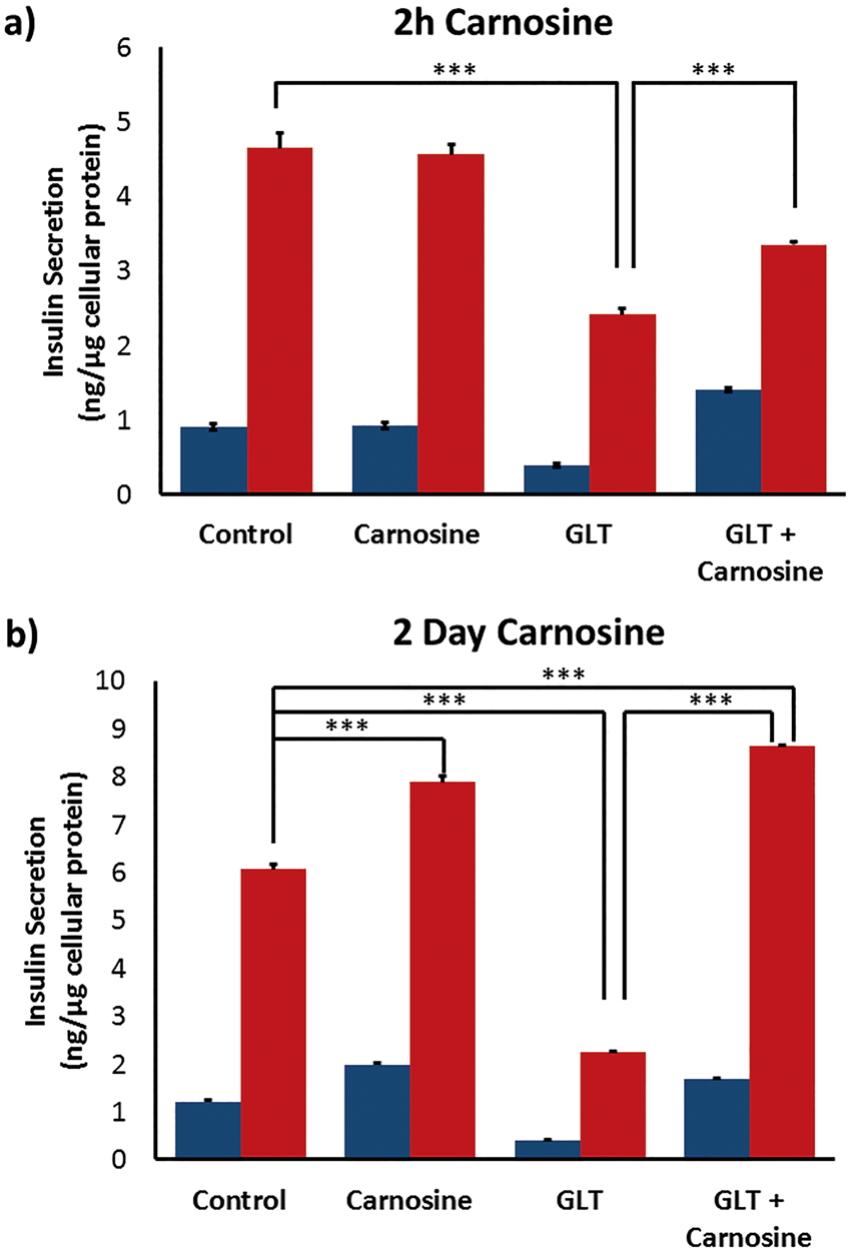
INS-1 cells were cultured in RPMI-1640 media, or RPMI GLT media for 5 days. Media were supplemented ± 10 mM carnosine for (**a**) 2 hours, or (**b**) 2 days prior to stimulation. Insulin secretion was determined by ELISA assay following incubation ± secretagogue cocktail for 2 h [(−) blue, (+) red], with data normalized to cellular protein content. Data are expressed as mean ± SEM from 3 independent experiments. ***p < 0.0005, with absolute values as stated in the main text.

Glucolipotoxicity is a hallmark of T2D, and we have previously shown that 72 h incubation of INS-1 cells in GLT media inhibited insulin secretion down close to basal levels^7^. Here we extended the incubation time to 5 days in order to maximise potential beneficial protective effects, but otherwise repeated these experiments to investigate whether carnosine would be able to reverse the inhibition of insulin secretion caused by GLT. We added carnosine to GLT media either 2 h (Fig. 3a) or 2 days (Fig. 3b) prior to secretagogue stimulation. The former resulted in a modest but statistically significant increase in insulin secretion. Addition of carnosine to INS-1 cells for the final 2 days of GLT media incubation was able to completely reverse the inhibition of insulin secretion caused by GLT. This indicates that not only is carnosine able to enhance insulin secretion, but that it can reverse the damaging inhibition in insulin secretion that results from chronic exposure to a high glucose and fatty acid environment.

### Carnosine Enhances Skeletal Muscle Glucose Uptake

**Figure 4:**
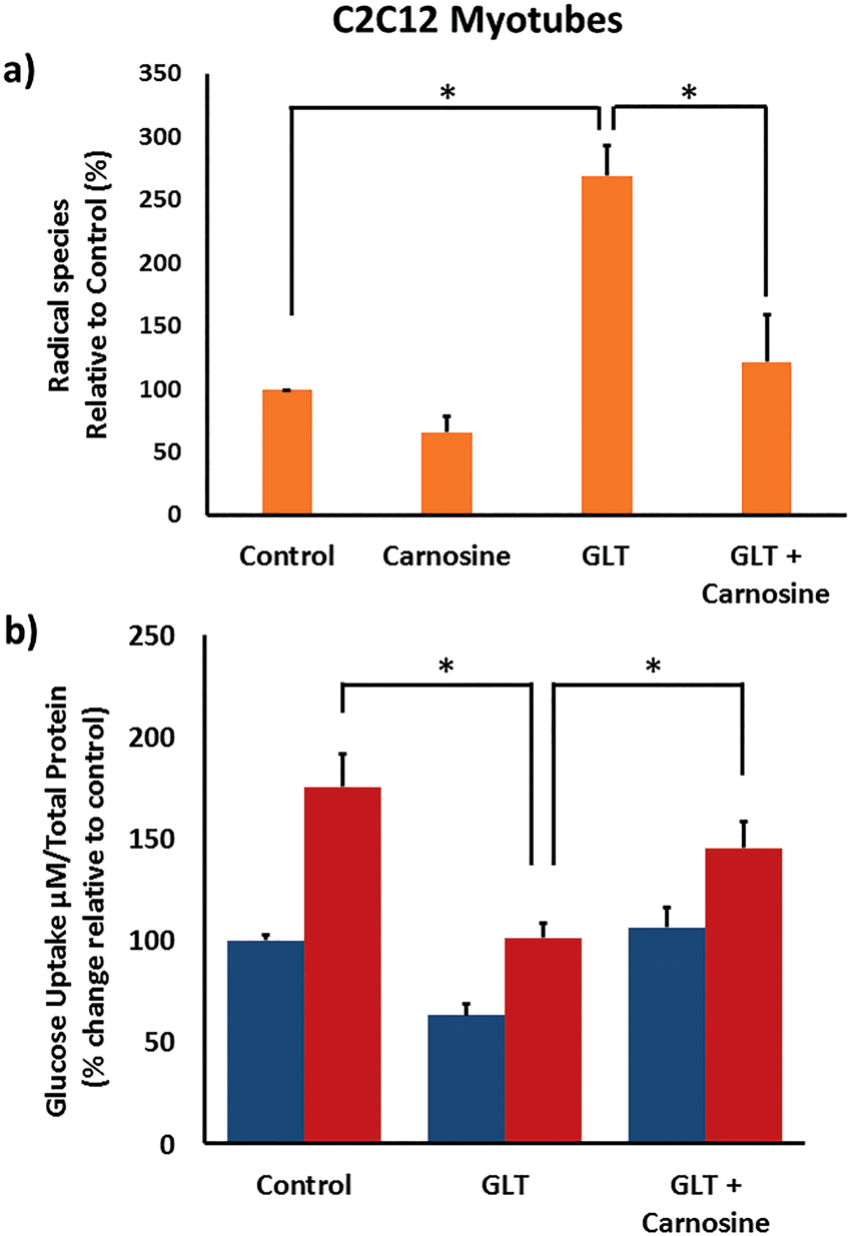
C2C12 myotubes were cultured in DMEM media, or DMEM GLT media for 5 days. Cells were then incubated for 1 h with media supplemented ± 10 mM carnosine. (**a**) 20 µM 2′,7-dichlorofluorescin diacetate was then loaded for 1 h and ROS detection measured via fluorescence with excitation and emission of 495 nm and 530 nm. ROS is expressed as percentage change relative to control. (**b**) Cells were serum-starved overnight in DMEM supplemented with 5 mM glucose, then incubated for 1 h in glucose-free DMEM +/−100nM insulin [(−) blue, (+) red]. Medium was replaced with PBS + 0.125 mM 2-deoxy glucose (2-DG). Glucose uptake reactions were conducted for 30 min. 2DG6P was detected using a luminometer. Data are expressed as means ± SEM of 3 or more independent experiments. *p < 0.05 **p < 0.005, with absolute values as stated in the main text.

Whilst failure of insulin secretion leads to overt T2D, control of glucose homeostasis also involves other cells and tissues including skeletal muscle. Moreover, peripheral insulin resistance often characterises pre-diabetes and involves failure of skeletal muscle to remove glucose from the bloodstream in response to insulin. We therefore sought to determine whether carnosine might also have a beneficial action on ROS scavenging and glucose uptake from skeletal muscle cells. C2C12 myotubes were cultured for 5 days in Dulbecco’s Minimal Eagle’s Medium (DMEM) media, or DMEM supplemented with 28 mM glucose and 200 μM palmitic acid and 200 μM oleic acid (GLT media). Cells were incubated+/−carnosine for 1 h and ROS scavenging determined using DCFDA as before. We observed a significant increase in ROS of 169 ± 23.9% (*p* = 0.002) in cells exposed to GLT media relative to control. Importantly, carnosine addition resulted in 87.0 ± 37.1% (*p* = 0.028) scavenging of GLT-mediated ROS species (Fig. 4a). This then resulted in a statistically significant increase in glucose uptake (*p* = 0.04) (Fig. 4b). As was the case with INS-1 cells, this is not due to significantly altered C2C12 cell viability, as in C2C12 myotubes treated with GLT media for 5 days, cell viability was found to be 93.67+/−3.61% (p > 0.05). Therefore carnosine scavenging is likely to exert a beneficial action on glucose homeostasis through both enhanced insulin secretion and skeletal muscle glucose uptake.

## Discussion

The damaging effects of glucolipotoxicity have been attributed to multiple biochemical consequences, including oxidative stress and non-enzymatic glycation with formation of advanced glycation endproducts (AGEs)^11^. AGEs are heterogeneous compounds that accumulate in sera and tissues of individuals suffering from diabetes and its complications^12,13^. Increased AGE formation occurs due to generation of RCS that react with amino acid residues on proteins to generate stable adducts^12,14,15^. In addition, glycation-derived free radicals can cause protein fragmentation, as well as oxidation of nucleic acids and lipids^16^ that change the physical properties of the recipient protein or lipid. This typically has a negative impact on their normal cellular function. Consistent with this hypothesis our data shows that GLT media generates reactive species, including peroxynitrite that is formed by superoxide combination with NO and results in 3-NT protein adduct formation. Similarly we find that GLT leads to damaging 4-HNE adduct formation, an α,β-unsaturated hydroxyalkenal that is most likely produced following peroxidation of intracellular lipids.

By manipulating molecules and pathways central to the pathophysiology of T2D, it might be possible to offer an improved clinical prognosis to significant numbers of patients suffering from diabetes. Therapeutic strategies aimed at reducing RCS- and AGE-induced tissue injury, including quenching of RCS by carbonyl scavengers, have previously been proposed and tested successfully in experimental animals^14^. Carnosine is a histidine-containing dipeptide anti-oxidant that serves as a major endogenous quencher of RCS via intramolecular Michael addition^17^ and is therefore a promising candidate as a therapeutic agent. This could potentially offer particularly high levels of protection in T2D, as significantly decreased levels of carnosine have been found in the kidneys^18^ and cardiac muscle^19^ in mouse models of diabetes, and in human skeletal muscle of type 2 diabetes patients^20^. Some of these findings are however controversial, and future studies might therefore seek to generate carnosine synthase (CARNS1−/−) knockout animals to provide a tissue carnosine depleted model, carnosinase-1 (CNDP1−/−) knockout animals to provide a plasma carnosine full model, and carnosinase-2 (CNDP2−/−) knockout animals to provide a tissue carnosine full model. By determining the prevalence to T2D in each of the above strains relative to control animals it might be possible to resolve this controversy.

A small group of enzymes are specifically suited to the detoxification and removal of 4-hydroxynonenal (4-HNE) from cells. Within this group are the glutathione S-transferases hGSTA4-4 and hGST5.8, aldose reductase, and aldehyde dehydrogenase. These enzymes have low Km values for HNE catalysis and together are very efficient at controlling the intracellular concentration. Unfortunately, however, pancreatic β-cells possess exceptionally low levels of glutathione enzymes^21^. This therefore renders them particularly susceptible to damage from 4-HNE. In order to offset the damaging effects of AGE and advanced lipidation products, other molecules are therefore needed in order to preserve β-cell function. Our data shows that carnosine is an effective species scavenger, both of the RONS that are the building blocks for synthesis of RCS, and of damaging adducts such as 3-NT and 4-HNE. By so doing, we have shown significant enhancement to the primary function of pancreatic β-cells, namely enhanced insulin secretion. Furthermore, in addition to a potential role for carnosine in increasing insulin secretion from the pancreas we have also demonstrated an increase in the uptake of glucose into C2C12 skeletal muscle cells, albeit further studies are needed in order to confirm these results using primary human cells.

In conclusion, data presented here indicates that carnosine is a highly effective scavenger of RONS, resulting in beneficial actions on glucose homeostasis through both increased insulin secretion and skeletal muscle glucose uptake. Carnosine supplementation as a therapeutic strategy might, however, require regular administration of high dosages of carnosine, as rapid turnover of carnosine occurs through the plasma enzyme carnosinase 1. Alternatively, if carnosine synthase, and transporters for the uptake of β-alanine and histidine, were present in the pancreatic β-cell, as they are in the skeletal muscle cell, then the β-cell would be in a position to synthesize its own carnosine. Under these circumstances increasing the dietary intake of either β-alanine or carnosine would be effective in increasing pancreatic content, although this is yet to be proven. Failing this, a more effective strategy may be to develop pharmacological non-hydrolysable carnosine analogs. Such compounds would not be turned over rapidly and should therefore offer more sustained scavenging potential, thereby enhancing insulin secretion with high efficacy at relatively low concentrations. These strategies will be the focus for future research in this area,

## Materials and Methods

### Materials

Antibodies were obtained from Abcam (Cambridge, UK) and Agilent Technologies (Santa Clara, CA, USA). Unless otherwise stated, all other chemicals were purchased from Sigma Aldrich (St. Louis, MO, USA) or VWR International Ltd (Lutterworth, UK).

### Islet Isolation and INS-1 β-Cell Culture

Islets were isolated from male CD1 mice by collagenase injection into the pancreatic duct. Digested pancreas was washed with MEM-2279 and separated from exocrine tissues by centrifuging through a Histopaque 1.077 g/ml gradient. After washing, islets were picked and incubated at 37 °C in RPMI-1640 (supplemented with 10% [vol/vol] fetal calf serum, 2 mM glutamine and 100U/ml penicillin/ 0.1 mg/ml streptomycin) for 24 h prior to further analysis. Rat INS-1 β-cells were cultured in RPMI-1640 media, or RPMI media supplemented where indicated with 28 mM glucose, 200 μM oleic acid, or 200 μM palmitic acid (GLT media) for 72 h as detailed previously^7^. All animal procedures were approved by the King’s College London Ethics Committee and carried out in accordance with the UK Home Office Animals (Scientific Procedures) Act 1986.

### C2C12 Cell Culture and Treatment

Mouse C2C12 skeletal myoblasts were maintained in high glucose-DMEM supplemented with 10% (v/v) fetal bovine serum, 10% (v/v) heat inactivated newborn calf serum (Life Technologies, Paisley, UK), and 1% (v/v) penicillin-streptomycin (Life Technologies) in a humidified atmosphere with 5% CO_2_ at 37 °C. Cells were switched to DMEM supplemented with 2% (v/v) heat-inactivated horse serum (Life Technologies) for 7 days in order to facilitate myocytic differentiation. Cells were then incubated for a further 3 days+/−GLT media and carnosine as indicated.

### Reactive Species Detection

INS-1 and C2C12 cells were cultured for 5 days in standard tissue culture media, or media supplemented with 28 mM glucose, 200 μM oleic acid, and 200 μM palmitic acid (GLT media). 10 mM carnosine was added and incubated for 1 h. Cells were washed 3 times in KRB, then 20 μM DCFDA loaded for 1 h. Radical species detection was measured via fluorescence, with excitation at 495 nm and emission at 530 nm. 3-NT residue formation was determined by ELISA (Abcam) and absorbance read at 450 nm. 4-HNE formation was determined by ELISA (Abexxa) and absorbance read at 450 nm. In all cases radical species are expressed as percentage change relative to control.

### Cell Viability

INS-1 and C2C12 cells were cultured in RPMI 1640 or GLT media for 5 days before media was aspirated and cells washed 3 times in KRB. A final concentration of 5 µM Calcein AM Cell Viability Dye (ThermoFischer) in KRB was loaded for 1 hour before washing again with KRB. Cell viability was measured via fluorescence, with excitation and emission at 490 nm and 520 nm respectively.

### Western Blotting

INS-1 cells were lysed and protein separated by SDS-PAGE, then transferred to nitrocellulose as described previously^7^. Protein was detected using anti-iNOS (Abcam, Cambridge UK) primary antibody and polyclonal goat anti-mouse horseradish peroxidase conjugated secondary antibody (Agilent Technologies, Santa Clara, CA, USA).

### Insulin Secretion

INS-1 cells were treated with KRB or KRB supplemented with secretagogue cocktail (13.5 mM glucose, 1 μM phorbol 12-myristate 13-acetate, 1 mM isobutyl-methylxanthine, 1 mM tolbutamide, 10 mM leucine, 10 mM glutamine) for the indicated time. Insulin secretion was determined using ELISA kit as detailed previously^7^. Size-matched islets were pre-incubated for 1 h at 37 °C in buffer containing 2 mM glucose, 2 mM CaCl_2_ and 0.5 mg/ml BSA, pH 7.4. Islets were further incubated in buffer containing 2 or 20 mM glucose for 1 h at 37 °C with gentle shaking. Insulin secretion was measured by radioimmunoassay with an in-house ^125^I-labelled insulin tracer as detailed previously^22^.

### Glucose Uptake

Following the indicated treatment, cells were serum-starved overnight in DMEM supplemented with 5 mM glucose, then incubated for 1 h in glucose-free DMEM +/−100nM insulin. Medium was replaced with PBS + 0.125 mM 2-deoxy glucose (2-DG). Glucose uptake reactions were conducted for 30 min, and then terminated by addition of stop buffer (0.4 M HCl + 2% dodecyl trimethyl ammonium bromide). 2DG6P detection reagent was applied and data were acquired using a CLARIOStar luminometer (BMG Labtech, Ortenberg, Germany).

### Statistical Analysis

Results are expressed as mean ± standard error of the mean (n = 3 or more independent experiments). Parameters were compared using unpaired student t-test, with statistical significance determined using Holm-Sidak method with an alpha value of 5%, and analysing samples individually assuming consistent standard distribution. A *p* value below 0.05 was considered to be statistically significant.

## Electronic supplementary material

Supplementary Figure 1
