# Supplementary material for: Carnosine scavenging of glucolipotoxic free radicals enhances insulin secretion and glucose uptake
Source: Sci Rep. 2017 Oct 17;7:13313. doi: 10.1038/s41598-017-13649-w (PMC5645413; doi:10.1038/s41598-017-13649-w)
Supplement: Supplementary file 1 — Supplementary Figure 1 [file 41598_2017_13649_MOESM1_ESM.pdf]

Carnosine scavenging of glucolipotoxic free radicals enhances insulin secretion and glucose uptake.

Michael J. Cripps[1], Katie Hanna[1], Charlie Lavilla[1], Sophie R. Sayers[2], Paul W. Caton[2], Craig Sims[1], Luigi De Girolamo[1], Craig Sale[3], and Mark D. Turner[1].

[1]Interdisciplinary Biomedical Research Centre, School of Science and Technology, Nottingham Trent University, Clifton, Nottingham, NG11 8NS, UK.. [2]Diabetes and Nutritional Sciences Division, King's College London, London, United Kingdom, SE1 1UL, [3]Sport, Health and Performance Enhancement (SHAPE) Research Centre, School of Science and Technology, Nottingham Trent University, Clifton, Nottingham, NG11 8NS, UK..

*Correspondence to:
Dr. Mark D. Turner,
Interdisciplinary Biomedical Research Centre.
School of Science and Technology,
Nottingham Trent University,
Clifton Road,
Nottingham, NG11 8NS
United Kingdom.

E-mail: mark.turner@ntu.ac.uk

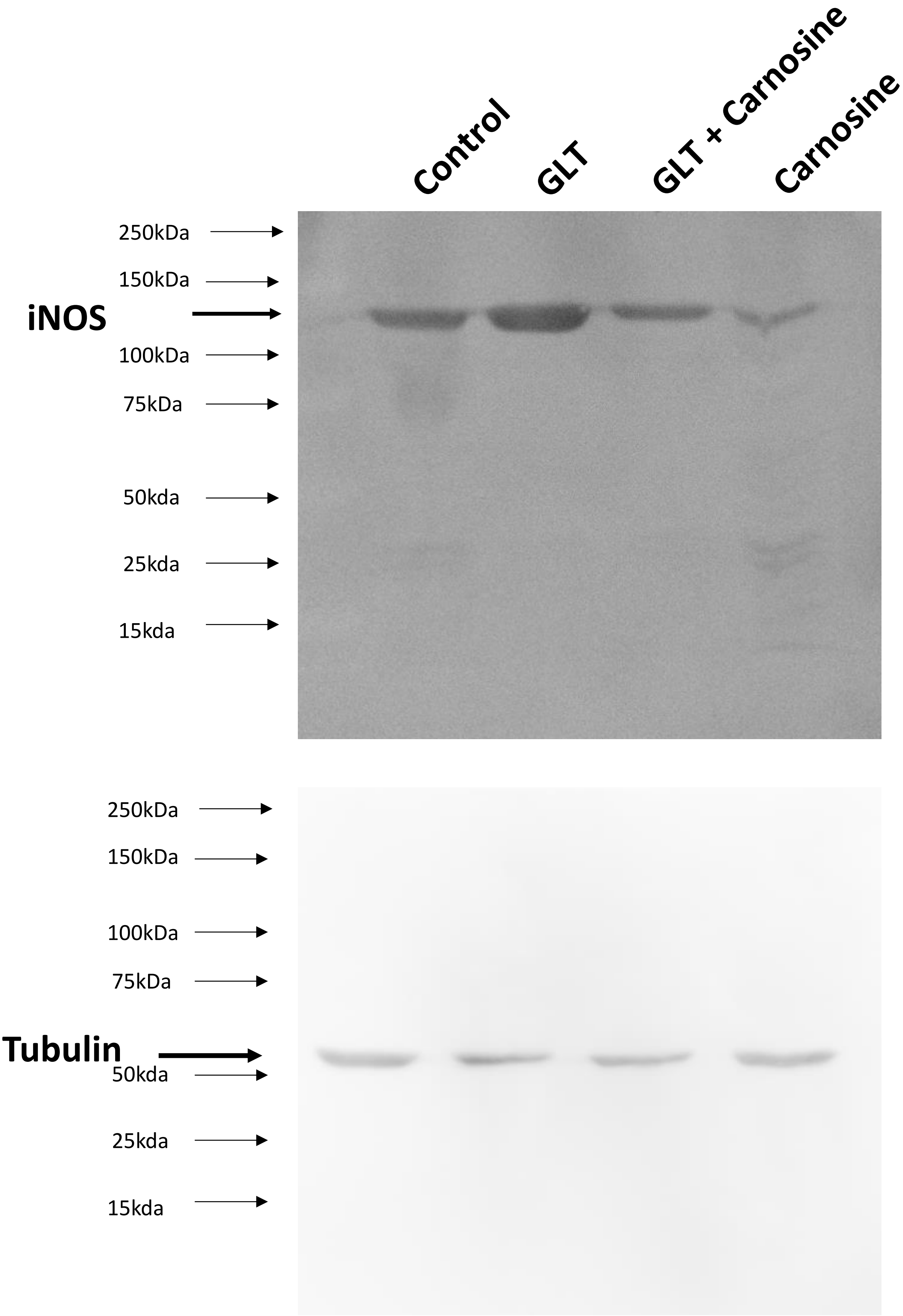
Control
GLT
GLT + Carnosine
Carnosine
250kDa
150kDa
iNOS
100kDa
75kDa
50kda
25kda
15kda
250kDa
150kDa
100kDa
75kDa
Tubulin
50kda
25kda
15kda

Supplementary Figure 1

INS -1 cells were cultured in RPMI-1640 media, or RPMI GLT media for 5 days ± 10mM carnosine. Protein was separated by SDS-PAGE, transferred to nitrocellulose, and specific proteins detected using either anti-iNOS or anti-tubulin primary antibody.
